# Reflexive pedagogy at the heart of educational digital transformation in Latin American higher education institutions

**DOI:** 10.1186/s41239-022-00365-3

**Published:** 2022-10-25

**Authors:** Ana Carolina Useche, Álvaro H. Galvis, Frida Díaz-Barriga Arceo, Alberto Elí Patiño Rivera, Claudia Muñoz-Reyes

**Affiliations:** 1 Pontificia Universidad Javeriana, Bogotá, Colombia; 2 Universidad de Los Andes, Bogotá, Colombia; 3 Universidad Nacional Autónoma de México, México City, México; 4 Pontificia Universidad Católica del Perú, Lima, Perú; 5 Stanford University, Stanford, CA USA

**Keywords:** Active learning, Artificial intelligence, Curricular transformation, Digital transformation, e-Portfolio, Gamification, Higher education, Latin America, Networked learning, Post-pandemic teaching, Reflexive pedagogy, Robotics

## Abstract

This paper makes a critical review of educational and operational issues related with pandemic and postpandemic lessons in Latin American higher education institutions (LATAM HEI), as background for uncovering key elements to innovate educational practices in technology-mediated higher education. The authors adapted the reflexive pedagogy framework to conduct in depth analysis of innovation experiences mediated with educational technologies and draw conclusions for curricular and digital transformation of LATAM HEI.

## Introduction

The emergence of the COVID-19 pandemic in 2020 led universities all over the globe to send their students home and implement emergency remote classes. Teaching was carried out partially or totally online and mostly without planning. In 2021, higher education institutions, better prepared to face the pandemic, implemented different teaching modalities (hybrid, online, etc.). At the beginning of the crisis, the first response of many educational institutions in Latin America was to invest in learning management systems and technological tools that would enable them to continue teaching and learning during the pandemic. As faculty, students and administrative staff became better acquainted with the technological tools, questions related to the evaluation of student learning, and to the implementation of more active and collaborative learning approaches became the focus of attention. In this article, we elaborate on the issue of why a reflexive pedagogy needs to be at the heart of a digital transformation in Latin American higher education organizations. As illustrated by the four articles commented in this special issue, this framework enables educational innovations supported by technology to be effectively implemented. The purpose has been to document good educational practices with the mediation of digital technologies, while recognizing the digital divide that operates in our region and the low level of digital literacy.

## Latin-American context

The highly unequal income distribution across the Latin-American population is one key characteristic that differentiates this region from others in the world. The pandemic prompted an increase in poverty and inequality that, in the presence of weak and inefficient social protection policies and health systems, worsened (or has the potential to worsen) social conflict (Currea & Hidalgo, 2021).

The pandemic evidenced the vulnerability of many students in higher education institutions. The economic impact, health concerns and lockdowns resulted in students’ mental health issues such as anxiety and depression (Reimers, 2021). Women were affected disproportionately due to a gender-differentiated access to internet and devices, as well as by the fact that they carry a greater proportion of the burden on domestic activities. Indeed, domestic violence against women increased because of the pandemic related lockdown measures (Ordorika, 2020). Low socioeconomic status students had less access to broadband services and computers, were less likely to have competencies for e-learning and were more likely to drop out of college because of the economic impact of the pandemic (Area-Moreira et al., 2021; Defelipe, 2021; Reimers, 2021; UNESCO IESALC, 2021).

These difficulties became a concern to faculty and administrative staff and resulted in universities having to carry the burden of socioeconomic situations far from their reach (Arrufat, 2021). Making things worse, universities were, at the same time, facing diminishing investment of public funds in education, with increasing costs due to investment in technological tools and platforms, acquisition of computers and software, and adaptation of classrooms. In parallel, adoption of biosecurity protocols, as well as shortages of human resources and diminishing student enrollment were in place (Abadía et al., 2020; Agencia de Noticias Univalle, 2021; Ordorika, 2020).

## Reflexive pedagogy

Remote teaching and learning technologies were important during the sanitary emergency, and reflection on them can provide important lessons. These technologies enabled students to receive lessons from their home (*learning anywhere and anytime*) with the support of diverse educational resources such as texts, graphics, audios, videos, simulations etc. (*learning supported by different media*). Initially, students were receiving lectures about the content of the class for a big part of the day. Soon, it became evident that students couldn’t apprehend endless content transmission and raised concerns about how to make learning a more active experience. In consequence, instructors began introducing activities in which students could actively make meaning of the content that was being delivered through different media and learning platforms (*active knowledge making*). The former, led to emphasize different teaching methods, for example, inquiry-based teaching methods, such as problem-based learning, case-based learning, project-based learning, etc.

Additional learning difficulties were posed by the fact that students were not able to go to campus and meet their peers and professors, which limited their possibilities of social and academic interaction, underlining the need to design and implement collaborative learning approaches (*collaborative learning*).

Remotely assessing students’ knowledge proved to be difficult, since students had unrestricted access to contents in their home environment. The latter led to concerns of grade inflation and accentuated the need to change testing subjects and methods. Testing what students can do with the content as opposed to testing what they remember about it has become a new priority (*summative vs. formative assessment and critically reflecting on their learning process*).

As mentioned before, the pandemic particularly evidenced the struggles of the most vulnerable students, making it a priority for faculty to carefully confront each individual student situation, and highlighting the need of personalized approaches to effectively respond to student needs (*differentiated learning*).

The pandemic evidenced that to promote student learning and well-being mediated by technologies, higher education institutions cannot focus only on digitalizing content and delivering it to students (*emergency remote teaching*). On the contrary, institutions need to focus on developing learning environments that integrate the possibilities that technologies afford (*learning anywhere and anytime and supported by different media)* with student’s active knowledge making, formative assessment, collaborative learning, critical self-reflection on their learning process and differentiated learning (Galvis & Carvajal, 2022).

Challenges as the above, derived from the forced and rapid adoption of educational technologies in Latin-American Universities due to the pandemic, put the need to adopt reflexive pedagogies at the forefront of academic programming needs in the region, in contrast with conventional didactic pedagogies that both faculty members and students used to consider “normal” (Graham & Robinson, 2007).

The following table contrasts in seven dimensions the principles of didactic versus reflexive pedagogy as proposed by Cope and Kalantzis (2017). In addition, the editorial team of the ETHE-LATAM special issue commented these seven dimensions. We chose this model as a reference for the design and analysis of educational innovations because it reflects many of the concerns of the papers received for this collection and articulates them in a single multidimensional model (Table 1).

**Table 1:** Multidimensional comparison of didactic and reflective pedagogies. Source: Adapted from (Cope & Kalantzis, 2017) by the ETHE-LATAM editorial team

Dimension	Didactic pedagogy	Reflexive pedagogy	Editorial team comment
*Spatio-temporal dimension*	Confined by the four walls of the classroom and cells of the timetable	Ubiquitous learning: anywhere, anytime, anyhow	The transition from face-to-face or in person educational interaction, to technology-mediated synchronous and asynchronous educationally oriented communication, adds flexibility to when, how and where to learn
*Epistemic dimension*	The learner as knowledge consumer, passive knowledge acquisition, memorization	Active knowledge making: the learner-as-knowledge producer and discerning knowledge discoverer/navigator	This dimension is at the heart of the educational transformation that can occur when moving from didactic to reflective teaching. It is, perhaps, the most challenging dimension since it entails changes in the place of control of the educational process: teacher-centered vs. student-centered. The teacher becomes the facilitator of the learning process, and the student actively constructs his/her understanding
*Discursive dimension*	Academic literacies: traditional textbooks, student assignments and tests	Multimodal meaning: new media texts, multimodal knowledge representations	Multiple representations of knowledge such as texts, diagrams, pictures, etc. can make abstract content easier to understand Also, students can produce their own ways of represent their understanding in multiple media and can share it with their peers and instructors. Digital platforms and media texts also allow to share their own learning materials and knowledge outside the walls of their classroom and university
*Evaluative dimension*	Emphasis on summative assessments and retrospective judgments that serve managerial purposes but are not immediately actionable	Recursive feedback: formative assessment, prospective and constructive feedback, learning analytics	Changes in the evaluative dimension recognize that assessment can be a means of learning and not just a means of demonstrating that it has been learned. These changes go beyond the type of grading system (numerical or A/R) and recognize the importance of evaluation for diagnostic and formative purposes With today’s digital innovative evaluation tools, it is possible to evaluate the learning process in real time. And it is in benefit not only for the instructor, but also serves as a self-evaluation tool for the student
*Social dimension*	The isolated learner, with a focus on individual cognition and memory	Collaborative intelligence: peer-to peer learning, sourcing social memory and using available knowledge tools appropriately	The transformation from the social dimension perspective makes it evident that interaction with others, peers and teachers is a mean to reach knowledge and to socialize and enrich the cultural, scientific, and technological heritage Besides that, collaborative learning promotes skills such as critical thinking and leadership, which ultimately enhance the whole formative process
*Cognitive dimension*	Focus on facts to be remembered, theories to be correctly applied	Metacognition: thinking about thinking, critical self-reflection on knowledge processes and disciplinary practices	Learning to learn and to go beyond what has been learned enriches capacities to plan, monitor, control and reflect on what has been learned
*Comparative dimension*	Homogenizing, one-size-fits-all curriculum, standardized teaching and assessment	Differentiated learning: flexible, self-expressive and adaptive learning, addressing each student according to their interests, self-identity and needs	The changes in the comparative dimension deal with academic flexibility, both in the curricular (for what and what to learn), pedagogical (how and with what to learn), administrative (in what modality and with what certification, if applicable)

## On this issue

“Technology-mediated educational innovations in Latin American Higher Education institutions” was the focus of the ETHE-LATAM call for papers launched in April 2021 by the *International Journal of Educational Technology in Higher Education* (ETHE). The invitation aimed at addressing issues that affect Latin American higher education communities locally, regionally, or globally. This initiative intended to foster dissemination of evidence-based educational innovations that make a difference in their context and build new opportunities upon technology-based avenues.

By the end of August 2021, at ETHE-LATAM special issue closure, the board of editors had received 27 original works. Because lack of alignment with the call, five papers were redirected to the Springer transfer desk, with notification to corresponding authors. Remaining papers were submitted for peer review; two or three ETHE referees provided feedback to the corresponding author and to the invited editors, who made final decisions, based on reviewers’ recommendations.

This position paper emerges from the review of literature and experiences related with higher education digital transformation as well as from the analysis of tensions and trends that may have impact on the “back to normal”, i.e., postpandemic situation, in Latin America. Digital technologies became part of the educational scenario at all educational levels when remote teaching turned into a need-to-be condition; they *enabled* content management as well as distributed interaction among educational agents but did not provide a fully educational solution. The transformational potential of technology-mediated blends (Graham & Robinson, 2007) became apparent, as the educational community realized that it was possible to *enhance* the educational experience by implementing student active learning facilitated by the instructor in collaboration with peers. The literature review brought us to *reflexive pedagogy* (Cope & Kalantzis, 2017), a multidimensional perspective that may help designing and assessing technology-mediated educational innovations.

The four cases selected for this collection studied the use of different technologies in higher education. In alphabetical order per topic, the four papers deal with (1) artificial intelligence applications in education, (2) e-Portfolio and networked learning in ADS—architectural design studio, (3) gamification and collaboration for STEM—science, technology, engineering, and math—teaching, (4) robotics and experiential learning in STEM courses. With the following resemblance, we hope the reader will be motivated to read each paper and to reflect on the value reflexive pedagogy can add to her/his own experience.

### Artificial intelligence applications in education in Latin American higher education: a systematic review

The analysis conducted by Salas-Pilco and Yang (2022) is a systematic review of the literature on the use and applications of Artificial Intelligence—AI—in higher education institutions in Latin America. The authors reviewed articles published between July 2016 and June 2021, finding in total 2397 studies. Only 31 studies met all the criteria for the analysis.

The review answered the question: What and how are AI-based applications being used by higher education institutions in Latin America? The results revealed that the main AI applications refer to: predictive modeling (e.g., predict students’ dropout rates, course and academic performance); intelligent analytics (e.g., identify predictors of university graduates’ employability, analyze the impact of universities classified as accredited or non-accredited on student standardized tests, and evaluate academic research performance and scientific productivity); assistive technology (e.g., chatbots that identify students with mental health issues); automatic content analysis (e.g., student online assessment, extracting information from university documents to generate a dataset that can provide answers to queries, evaluating teacher performance through student comments); and image analytics (e.g., managing student attendance using facial recognition).

In addition, the review attempted to answer the question: What educational issues and problems are being addressed by AI applications in Latin American higher education institutions? The results were organized in three categories: AI applications to (1) support student learning, (2) to help teachers to offer quality education and (3) to improve university services.

Concerning AI applications to support student learning, the review found AI applications to study the factors that predict students’ academic performance and employability of graduates, and using chatbots to identify mental health issues. About AI applications to help teachers offer a quality education, the review found studies focused on using AI to analyze students’ evaluations of the course, to analyze other information produced by different educational activities, and to improve online assessment and teacher-student communication. Relating to AI applications to improve university services, the studies focused on studying the factors that predict dropout and retention, improving services to answer students’ questions or concerns (chatbots), and evaluate university performance for accreditation, governance, and research purposes.

Within the framework of reflexive pedagogy, AI offers a very wide range of possibilities for education. We highlight some of them.

- *Spatio-temporal dimension*: AI can contribute to ubiquitous learning by facilitating the construction of tools that make it easier for a student to receive personalized instruction anywhere and at any time.
- *Epistemic dimension*: No study was found that focused on the student as knowledge producer.
- *Discursive dimension*: No study was found that potentiated AI for multiple representations of knowledge.
- *Evaluative dimension:* AI enables the creation of evaluation systems focused on the learning process capable of detecting how students are responding to learning situations and the proposed activities and offering them feedback so that they deepen their successes or correct their mistakes. They then offer pathways and resources that correspond to their learning situation and are tailored to their needs and abilities.
- *Social dimension:* Through AI, shared environments can be built that facilitate the intercommunication and interaction of a community of students and teachers committed to a common task. An intelligent “agent” oversees and mediates the interaction to achieve the learning objectives.
- *Cognitive dimension:* No study was found that focused on the improvement of metacognition with AI.
- *Comparative dimension:* AI allows using intelligent systems to build and recommend personalized learning paths that adapt content and learning experiences to the individual potential of each student.

### e-Portfolio to promote networked learning in ADS

The study conducted by Roco Ibaceta and Barberá Gregori (2022) explored the use of e-Portfolios to promote networked learning in the context of the Architectural Design Studio (ADS). The ADS is a socially active environment of experimentation and collaboration between students who, individually or in small teams, are invited to present the development of their projects in front of peers and expert audiences and are subjected to criticism by their tutors or other guests.

Networked learning (NL) is defined as learning in which information and communications technologies promote connections between a learning community and its resources. Within the NL approach, the implementation of the e-Portfolio is presented as a tool with the potential to promote the communication and interaction of the members of an educational community, support the learning process and help learners recognize and develop their own learning trajectories.

Specifically, the authors explored the impact of the e-Portfolio on the application of eight networked learning principles (NLP). NLP include (1) support the learning process as perceived by the students; (2) promote shared responsibility for the learning process; (3) enable time to facilitate the construction of relationships; (4) promote situational and context dependent learning; (5) foster collaborative learning; (6) promote dialogue and social interaction to support the construction of knowledge, identity and learning; (7) foster critical reflexivity as an important part of the learning and knowledge process; (8) be sure that the facilitator has a relevant role in the networked learning. Results showed a positive increase on 7 of the 8 NLP. The e-Portfolio implementation particularly supported critical reflection, dialogue and social interaction, and student perception that the e-Portfolio supported their learning process.

In addition, the authors investigated the support of the e-Portfolio to four dimensions of the training process in ADS: (1) the teaching strategy (e.g., assessment of student’s work); (2) the training context (e.g., development of teamwork); (3) the construction of disciplinary learning (e.g., development of a technical reference framework on architecture) and, (4) projection of the training process (e.g., use the e-Portfolio in other workshops in the career). Findings showed that the teaching strategy and projection of the training process dimensions were particularly supported by the e-Portfolio.

Looking at this educational innovation with the reflexive pedagogy lens, it can be stated that:

- *Spatio-temporal dimension*: The use of e-Portfolios to document and reflect on students’ work and academic progress gives spatio-temporal flexibility to the learning process.
- *Epistemic and social dimensions*: Majority of the eight NLP promote the student as knowledge producer and collaborative pedagogy.
- *Discursive dimension*: ADS is multimodal; students learn from a combination of modes enhanced with digital technologies.
- *Evaluative dimension:* e-Portfolios are formative assessment tools embedded in ADS teaching protocols.
- *Cognitive dimension:* e-Portfolios support critical reflexivity as part of the learning process and self-reflection of knowledge processes, principles 1 and 7 of the networked learning framework, as the results of this study show.
- *Comparative dimension:* Differentiated learning is a natural outcome from experiential (via ADS) and reflective (via NLP application) learning process.

### Gamification and collaboration for STEM teaching

The main goal of the paper: “Gamification suffers from the novelty effect but benefits from the familiarization effect: findings from a longitudinal study” (Rodríguez et al., 2022) is to understand how the impact of a gamification design, featuring fictional and competitive collaborative elements, changes over a 14-week period, when applied to CS1—Introductory Programming—courses taken by Brazilian STEM students.

The gamification’s goal was to motivate students to complete programming assignments. A quasi-experimental study allowed comparing teaching of CS1 with and without gamification to students from different STEM disciplines. Both groups had the same type of interaction with CodeBench©, a drill-and-practice system that delivers programming problems selected o created by instructors.

The gamification goal was to motivate students to complete programming assignments, either during face-to-face or online classes, when more suitable to the learners, but always using CodeBench©. To achieve that goal, the gamification allows the students to see themselves as characters from a medieval fantasy world (narrative). The students can customize these characters (i.e., their avatar) and, if they receive a high score in a series of assignments, their avatar gets a badge that leads to a more powerful weapon. Furthermore, the gamification design also enables students to join their classmates (collaboration) to fight a monster, aiming to free the fantasy world from it.

Results indicate changes in gamification’s impact that appear to follow a U-shaped pattern. Supporting the novelty effect, the gamification’s effect started to decrease after 4 weeks, decrease that lasted between 2 and 6 weeks. Interestingly, the gamification’s impact shifted to an uptrend between 6 and 10 weeks after the start of the intervention, partially recovering its contribution. These findings provide some guidelines to inform about both, the novelty effect, and the familiarization effect, concerning the real impact of gamification on some student’s behavior. The empirical evidence demonstrates the long-term effectiveness of an innovative gamification design, which features fictional and competitive-collaborative game elements, in improving positive learning behaviors.

This research study includes some dimensions of reflexive pedagogy, depending on course design and intervention:

- *Spatio-temporal dimension*: Flexible time and place was allowed for face-to-face and online student interaction with CodeBench© [control and experimental groups].
- *Epistemic dimension*: Active learning fostered via programming problem solving with immediate feedback was in place [control and experimental groups].
- *Discursive dimension*: Game design features provided opportunities for students to learn in a fictional environment with that media motivating students to practice problem solving [experimental group].
- *Evaluative dimension*: Problem solving with constructive feedback was a learning strategy; learning analytics were in place to help instructors make informed teaching decisions [control and experimental groups].
- *Social dimension*: Collaboration with classmates allowed to purposely fight a monster [experimental group].
- *Cognitive dimension*: The design did not include metacognition [control and experimental groups].
- *Comparative dimension*: The design did not include adaptive learning [control and experimental groups].

### Robotics and experiential learning in STEM courses

The study, developed by (Boya-Lara et al., 2022), evaluates the effects of designing, constructing and operating BEAM (Biology, Electronics, Aesthetics, Mechanics) robots in the development of knowledge and skills in electrical, electronics, and mechanic engineering. The robots were also used to develop computational thinking. An online course and its respective curriculum were designed and implemented to study this proposal. The course was presented to a sample of fifteen students from the engineering faculty of the Universidad Interamericana de Panama, and various data collection tools were applied.

The course had four phases. In the first phase, the student performed learning activities focused on recognizing, understanding, and interpreting concepts, definitions, terms, relationships, and procedures in electricity, electronics, and mechanics. The Robots Construction phase was focused on the application of those concepts and procedures, and the development of concrete skills such as electronic welding, interpretation of electronic diagrams, and concretization through the construction of the robot. The next phase sought to develop computational thinking and that students learn how to recycle Waste Electrical and Electronic Equipment (WEEE) to integrate them in the building of the robot. The final phase was intended to stimulate creativity through a robot design activity.

A pre- and post-survey was applied to the students to evaluate the impact of the course on five dimensions of STEM knowledge and skills: Electrical, Electronics, Mechanics, Computational Thinking, and recycling of WEEEs. A t-test evidenced a significant change in all STEM knowledge and skills in students, except computational thinking. Furthermore, all students were able to build the two robots and send videos showing the process of building the robot and explaining their reasoning. Nevertheless, the students were challenged to propose a new BEAM robot design, using the parts recovered from the WEEEs. Only seven out of fifteen students completed this activity.

Analyzing this learning innovation with the reflexive pedagogy model, we can observe the following characteristics of BEAM Robots construction in all categories, as follows:

- *Spatio-temporal dimension*: The complete course was taken on-line as the COVID pandemic did not allow for in-person instruction, allowing students to interact synchronously and asynchronously with their instructors and peers.
- *Epistemic dimension*: The students had to build two robots, identify their different parts and functions, abstract useful information from an electronic scheme and concretize it in a machine. In addition, students had to assess the behavior of the robot, identify errors and correct them. Furthermore, students were encouraged to develop alternative versions of the BEAM robots.
- *Discursive dimension*: The course used video guides for the various learning activities, brief papers or videos to explain the concepts of each topic of the module. In addition, assessment activities could have different formats (questionnaires, texts, videos) and students had to reference the written or multimedia material used to support their learning. Also, a kit with the parts and tools necessary to construct two robots was delivered to students.
- *Evaluative dimension*: Besides the assessment activities mentioned before, students had a logbook in which they had to reflect on lessons learned, experiences, problems, comments, criticisms, etc. Furthermore, at the end of each week, the instructors had a synchronous session with the students to clarify doubts, make comments, and receive more information.
- *Social dimension*: Each week students had a synchronous session in which they could interact with their instructors and peers. Also, in the last session, the students were asked to present their work to their tutors, peers, and some other invitees.
- *Cognitive dimension*: As an evaluation activity, the students were asked to explain the construction process and develop a robot's operating procedure flowchart. In addition, students had to identify and solve troubleshooting problems and errors that prevent the robot to work as it should. Also, students had to assess whether it was possible to use the parts recovered from the WEEEs in the re-design of the robot.
- *Comparative dimension:* This innovative course was completely flexible from its conception, since the lecturers could not meet their students, the kits containing the robots’ parts and even the recyclable parts to be extracted from electronic waste and integrated to the robots were delivered to the student’s home for them to construct and present it at the end of the course.

## Conclusions

The articles selected for this ETHE-LATAM issue show educational technologies that emerged since the introduction of computer-mediated and online learning. Nevertheless, these technologies cannot be considered new. The possibility of using artificial intelligence to teach began in the mid-1980s with a focus on teaching arithmetic (Bates, 2015). E-Portfolios had a boom in the early 2000s and were adopted long before that (Lam, 2020). Gamification has been used as a construct since 2010 (Koivisto & Haman, 2019). The interest in robotics on educational settings has been discussed at least since 1993 (Papert, 1993).

However, we are still realizing the potential of these technologies on education. As the analysis of the articles of this issue presented, *we cannot understand the use of these technologies without a pedagogical framework that orients its use and potentialities for student learning*. Technologies are tools to support teaching and learning that in themselves do not communicate or create meaning (Galvis, 2021). For technologies to become a media for teaching and learning there must be a specific educational intention, with an instructor or educators designing learning environments and choosing the meaning they want to convey and students actively constructing their understanding.

*Artificial intelligence* is described as the ability of computer systems to carry out operations that would normally require human intelligence and, more precisely, as the ability of machines to use algorithms, learn from data, and use what they have learned to make decisions as a human being would (Rouhianen, 2018). Salas-Pilco and Yang (2022) identified AI applications in Latin América that support learning, teaching, and administrative functions. According to their review, educators in Latin America have not explored yet the use of intelligent tutoring systems, adaptive learning systems, and intelligent collaborative learning systems. These AI systems are significant improvements in automated computational approaches to education that could promote students’ active knowledge making and critical self-reflection. For example, adaptive learning systems provide automated feedback cycles which prompt students to reflect on their learning and take decisions about their progression.

*E-Portfolios* differ from paper portfolios in their possibility to include multiple media for knowledge representation, the ease of collaborative work, and the possibility of sharing complete work. The article presented in this issue explores the potentialities of e-Portfolios to include multiple media of representation in the Architectural Design Studio which supports specially the teaching strategy and the construction of disciplinary knowledge. Additionally, the possibility of sharing complete work promotes the options to explore and develop connected scholarly interests with opportunities at the academic and professional levels. Furthermore, the authors with the support of the networked learning framework further develop in which ways e-Portfolios facilitate collaborative learning and network building.

*Gamification* offers the possibility of promoting ubiquitous and active learning, providing a structured environment to practice various skills and receive recursive and differentiated feedback on student learning, fostering collaboration and motivation for learning. This may be by dividing a large whole into sub-tasks with clear goals and providing direct feedback for accomplishments, reframing an activity by establishing a meaningful narrative, or by gathering a social community to provide support (Koivisto & Haman, 2019). The article of Rodríguez et. al. (2022) focuses on understanding how the impact of a gamification design, featuring fictional and competitive collaborative elements, changes over a 14-week period, when applied to introductory programming courses. In this context, gamification encourages practice and feedback about solution correctness. Rodríguez et. al. (2022) found a U-shaped pattern on the impact on students’ motivation to practice coding. These findings may provide some guidelines to inform practitioners about the timing of the implementation of gamification in a learning context.

*Robotics* have been particularly used to improve learning of concepts and skills development in STEM areas, however it is not the use of robotics per se but the careful consideration of the educational experience what produces the educational outcomes (for a review see Benitti, 2012). The study of Boya-Lara et. al. (2022) underlines the importance of carefully considering different educational dimensions in the design of the educational experience: the emphasis on students’ active learning through their involvement in the conceptualization, design and operation of the robots, the use of multiple modes of knowledge representation to promote student understanding, the fostering of metacognitive skills by having students explain the construction process and capacity to assess possible errors that prevent the robot to work and alternate designs of the robot. Nevertheless, further studies can focus on studying how to promote metacognitive skills in robotics since only some of the students were able to achieve this. Lastly, constant feedback of student’s experience was a key factor for students’ learning process.

This analysis presents the potentialities of these technologies; however, it also suggests the risks of utilizing these technologies if they are not implemented in learning environments where a pedagogy orients it’s use to promote students’ learning and socioemotional wellbeing. For example, AI can be used to monitor student attendance using facial recognition which pose ethical and privacy issues. UNESCO, in the document *Artificial intelligence and education: Guidance for policy makers*, points out that “The increasing use of new AI technologies in education will only benefit all humanity if, by design, it improves human-centered pedagogic approaches and adheres to ethical norms and standards. AI must be aimed at improving learning for all students, empowering teachers, and strengthening learning management systems” (UNESCO, 2021).

Furthermore, these technologies convey the promise of increasing access to higher education, but that promise will never be completely fulfilled if higher education institutions are not able of provide high-quality education for all Latin American students considering the huge inequalities among them. That is, if technologies are not used in a way that promotes active engagement of students in their learning, collaborative relationships with others, student reflection on their learning process, multiple learning trajectories and knowing how to learn anywhere, anytime and use different media, technologies become merely devices to reproduce content and we wouldn’t be equipping students with what they need to be able to apply their theoretical knowledge to practical endeavors in a multitude of settings inside and outside of the classroom.

It would be short-sighted to focus only on the educational characteristics of technologies. There are social, organizational, cost and accessibility issues also to be considered. Particularly, higher education institutions in Latin America need to undergo curricular and organizational changes to make reflexive pedagogy a reality.

### Curricular transformation

A change of pedagogy cannot be undertaken without a cultural and organizational change led by a curricular transformation (Díaz Villa, 2007). Latin-American traditional curricular projects in higher education institutions continue to be encyclopedic with an accent on the content and facts of the disciplines (Magendzo, 1986). Instead, Latin American curriculums need to move from unidisciplinary curriculums centered on content to student centered curriculums with an emphasis on active learning rather than on the passive acquisition of knowledge. Also, these curriculums need to focus on the assessment of competence rather than on the ability to retain and recall unrelated facts (Bas Vilizio et al., 2021).

From an international perspective, (Wehmeyer & Zhao, 2020) postulate that it is not only about renewing the curriculum, but about transforming the foundations of teaching and what we understand by learning. From the outset, rigid and prescribed study plans should be abandoned. It should be necessary to adopt a flexible model of self-determined learning and a system oriented to the autonomy of the student who goes through various projects and scenarios, develops capacities and interests around goals of social and sustainable development, safety, health, and emotional well-being. Creativity, managing uncertainty and change will be the common denominator, with the slogan of adaptable and inclusive education for all. Technologies will only make sense if they lead to breaking down the barriers of classrooms and purely expository teaching, offering unprecedented experiences of growth and development. It is necessary to abandon the pressure to pass rote and information recognition tests, and look for other evidence of performance, for which the prosumer profile, creative tasks and learning analytics begin to take on relevance.

### Digital transformation

The pandemic boosted the digitalization of higher education institutions. It revealed the potential opportunities and limits of implementing learning modalities. Digitalization is considered a strategic priority for European, North American and Latin American Universities. At the core of a digital transformation is the curricular and pedagogical transformation presented earlier. It implies transformations in policies, institutional guidelines, decision-taking structures, institutional assessment practices, resource management, system supports, faculty development programs, research guidelines, mental health programs, financial support, and recruitment practices (for a review of good practices implemented by European and North American Universities, see Cirlan & Loukkola, 2021; Stewart et al., 2021).

For instance, less centralized curriculums would mean giving more autonomy to academic programs to take decisions depending on their specific circumstances. Also, creating groups integrated by different institutional representatives to take decisions about the different modalities of teaching. Furthermore, adopting technological support for teaching, learning and administrative processes would require new institutional policies and guidelines. In addition, curriculums could be reviewed to select the essential contents and focus on key competences expected from their graduates. Besides, to implement a reflexive pedagogy, Faculty can have support from teaching centers in reflecting about the different dimensions of learning and its articulation in the design of learning environments.

## Data Availability

Not applicable.
